# *Pleurotus* Macrofungi-Assisted Nanoparticle Synthesis and Its Potential Applications: A Review

**DOI:** 10.3390/jof6040351

**Published:** 2020-12-09

**Authors:** Kanchan Bhardwaj, Anirudh Sharma, Neeraj Tejwan, Sonali Bhardwaj, Prerna Bhardwaj, Eugenie Nepovimova, Ashwag Shami, Anu Kalia, Anil Kumar, Kamel A. Abd-Elsalam, Kamil Kuča

**Affiliations:** 1 School of Biological and Environmental Sciences, Shoolini University of Biotechnology and Management Sciences, Solan 173229, India; kanchankannu1992@gmail.com (K.B.); prernabhardwaj135@gmail.com (P.B.); 2 Advance School of Chemical Sciences, Shoolini University of Biotechnology and Management Sciences, Solan 173229, India; anirai3024@gmail.com (A.S.); neerajtejwan@gmail.com (N.T.); 3 School of Bioengineering and Biosciences, Lovely Professional University, Phagwara 144411, India; sonali.bhardwaj1414@gmail.com; 4 Department of Chemistry, Faculty of Science, University of Hradec Kralove, 50003 Hradec Kralove, Czech Republic; eugenie.nepovimova@uhk.cz; 5 Biology Department, College of Sciences, Princess Nourah bint Abdulrahman University, Riyadh 11671, Saudi Arabia; AYShami@pnu.edu.sa; 6 Electron Microscopy and Nanoscience Laboratory, Punjab Agricultural University, Ludhiana 141004, India; kaliaanu@pau.edu; 7 School Bioengineering and Food Technology, Shoolini University of Biotechnology and Management Sciences, Solan 173229, India; kumaranil@shooliniuniversity.com; 8 Agricultural Research Center (ARC), Plant Pathology Research Institute, Giza 12619, Egypt

**Keywords:** oyster mushroom, application, antibacterial, anticancer, antioxidant

## Abstract

Research and innovation in nanoparticles (NPs) synthesis derived from biomaterials have gained much attention due to their unique characteristics, such as low-cost, easy synthesis methods, high water solubility, and eco-friendly nature. NPs derived from macrofungi, including various mushroom species, such as *Agaricus bisporus, Pleurotus* spp., *Lentinus* spp., and *Ganoderma* spp. are well known to possess high nutritional, immune-modulatory, antimicrobial (antibacterial, antifungal and antiviral), antioxidant, and anticancerous properties. Fungi have intracellular metal uptake ability and maximum wall binding capacity; because of which, they have high metal tolerance and bioaccumulation ability. Primarily, two methods have been comprehended in the literature to synthesize metal NPs from macrofungi, i.e., the intracellular method, which refers to NP synthesis inside fungal cells by transportation of ions in the presence of enzymes; and the extracellular method, which refers to the treatment of fungal biomolecules aqueous filtrate with a metal precursor. *Pleurotus* derived metal NPs are known to inhibit the growth of numerous foodborne pathogenic bacteria and fungi. To the best of our knowledge, there is no such review article reported in the literature describing the synthesis and complete application and mechanism of NPs derived from macrofungi. Herein, we intend to summarize the progressive research on macrofungi derived NPs regarding their synthesis as well as applications in the area of antimicrobial (antibacterial & antifungal), anticancer, antioxidant, catalytic and food preservation. Additionally, the challenges associated with NPs synthesis will also be discussed.

## 1. Introduction

The enormous impact of nanobiotechnology on almost all life forms has intrigued researchers globally [1]. In 1959, Richard Feynman introduced the theoretical concept of miniaturization, and, for the first time, provided hidden hints on nanotechnology (directing towards technology, using materials that have dimensions of approximately 1–100 nm) [2]. Nowadays, microorganisms (bacterium, fungi, including mushroom, yeast) and green plants are used for green synthesis of metallic nanoparticles [3].

*Pleurotus* mushrooms, commonly known as oyster mushrooms, belong to the family of genus *Pleurotus,* and they are edible and nutritious in nature [4]. Oyster mushrooms are readily available and naturally grow in nearly all latitudes, tropical and subtropical forestry, except Antarctica [5]. The worldwide geographical distribution of different *Pleurotus* species, with optimum growth temperature, is shown in Table 1. The primary role of the fruiting bodies of oyster mushrooms is to absorb amino acids, proteins, vitamin B (niacin, thiamine, and riboflavin), vitamin D, carbohydrates, and mineral salts (iron, calcium, and phosphorus) [6,7]. Additionally, they show important antifungal, anti-inflammatory, antibacterial, and immunomodulatory activities.

Moreover, they have the ability to reduce sugar and cholesterol levels in the blood [11]. *P. ostreatus* contains β-1,3-D-glucan and pectin, which are water-soluble gel-forming substances, having the ability to bind with bile acids. They inhibit cholesterol-bile micelle formation, cholesterol absorption, and endogenous synthesis, while increase the removal of plasma cholesterol by reducing the production and secretion of very low density lipoproteins (VLDL) [12]. The content of the nutrients depends on the nature, age, and size of the fungus, as well as their growing conditions [13]. Several nutrients, such as carbohydrates (40–46%), protein (20–25%), fiber (10–21%), and amino acids (20–41%) are present in considerable amounts, while the content of fat is very low, ranging from 10–20% of the dry matter. Fungi fruiting bodies are a rich source of micro- and macro-elements, such as sodium, magnesium, phosphorus, calcium, manganese, potassium, iron, copper, and zinc. Mushrooms of the genus *Pleurotus* have their own importance, more than the commercially employed basidiomycetes, because they possess better superior nutritional, gastronomic, and medical properties than mushrooms (which can be easily cultivated on a broad range of substrates) [14]. Nowadays, mushrooms show significant potential in metal nanoparticle (NP) synthesis and multifaceted applications [15].

In recent decades, several reports on mycogenesis derived NPs have been published [11]. However, the precise mechanisms of synthesis of mycogenic nanomaterials with variable size dimensions and topologies are not well understood (yet). Members of the fungi kingdom include various heterotrophic multicellular eukaryotic organisms. These microbes play an essential role in diverse ecosystems, particularly in the nutrient cycling paradigms. Fungi can be reproduced by both processes, i.e., sexually as well as asexually, and have shown symbiotic relations with bacteria and plants. Fungi groups mostly consist of mildew, mold, rust, yeast, and mushrooms [16].

The benefits and relevant use of fungal cells, such as NP factories, are attributed to the release of high amounts of extracellular enzymes that can serve as bio-reducing (as well as stabilizing) agents for NP synthesis. Moreover, fungal-derived NPs are much better than the bacteria-derived NPs. Constituents, such as enzymes and metabolites secreted by fungal cells, play an important role in synthesizing metal NPs, which reduce the toxicity of substances [15]. Ions are often liable for toxicity. When metal ions in solution are exposed to bacterial cells, they become uniformly distributed in the environment surrounding the bacterial cell, with no specific localization. In contrast, NPs that interact with the bacterial cell wall produce a focal source of ions through continuous release of ions, and cause enhanced toxicity to the cells [17]. Positively charged metal ions can easily bind with the fungal cell surface containing negative charge through electrostatic and cell receptor-specific interactions. Both types of intracellular (as well as extracellular) fungi could be used to synthesize NPs. Fungi exhibit high metal-binding capabilities in comparison to bacteria, and, hence, fungal biomass has gained the attention of researchers, for the production of NPs, at a large-scale. Various metal NPs, such as PdNPs, AgNPs, AuNPs, CuNPs, FeNPs, ZnNPs, TiNPs, and PtNPs could be synthesized using their oxides, nitrides, sulfides, and fungal biomasses [18].

NPs derived from fungal biomasses exhibit distinct optical, physical, and chemical properties, such as high quantum yield, excellent biocompatibility, high photostability, and adequate near-infrared (NIR) light-absorbing capacity, owing to which, they can be used in various chemical and medical fields, such as sensing, medicine, catalysis, and food packaging [19,20]. Oyster mushrooms mediate myco NPs by using spent mushroom substrate (SMS), and have medical importance towards many pathogenic microorganisms, as reported for the first time in 2007 [3].

To the best of our knowledge, many review articles, based on the synthesis of metal NPs derived from diverse sources and their applications, have been reported. Still, few review articles have discussed the synthesis of distinct metal nanoparticles (MNPs) and several applications of oyster mushroom derived NPs, and we attempted to fill the gap. In this article, we intend to discuss NP synthesis derived from oyster mushrooms, using various techniques and different applications. First section discuses the synthesis of nanoparticles by using intra-and extra-cellular methods. Later, different applications involving antioxidant, anticancer, antibacterial, and catalysis, with possible mechanisms of action, have also been discussed.

## 2. Green Synthesis of Metal-Based Nanoparticles Mediated by Genus *Pleurotus*

Fungal exploration and implications in the area of nanotechnology are very significant. In previous literature, it was reported that microorganisms, including bacteria, fungi, and yeast, could be used for the synthesis of metal NPs (metal = calcium, gold, silicon, iron, silver, lead, and gypsum) [21]. We observed that fungi have received immense attention owing to their metal bioaccumulation properties, to produce metal NPs [22]. The fungal material includes mycelia, polysaccharides, and proteins are used in the formation of metal nanoparticles; metal NPs of oyster mushroom species were synthesized using mineral salts [23]. Fungi have intracellular metal uptake capabilities and maximum wall binding abilities because they have high metal tolerance plus bioaccumulation abilities [24,25,26,27]. In comparison with other plants and microbes, the mycelia of fungi provides effective hold ability in the bioreactor, as well as in agitation and high flow pressure [28]. Moreover, fungi secrete extracellular enzymes in high amounts, leading to the massive production of enzymes [29]. Reduction of the enzyme, using both intracellular and extracellular ways, help in metal NP synthesis, nanostructure, and biomimetic mineralization [30,31].

**Figure 1:**
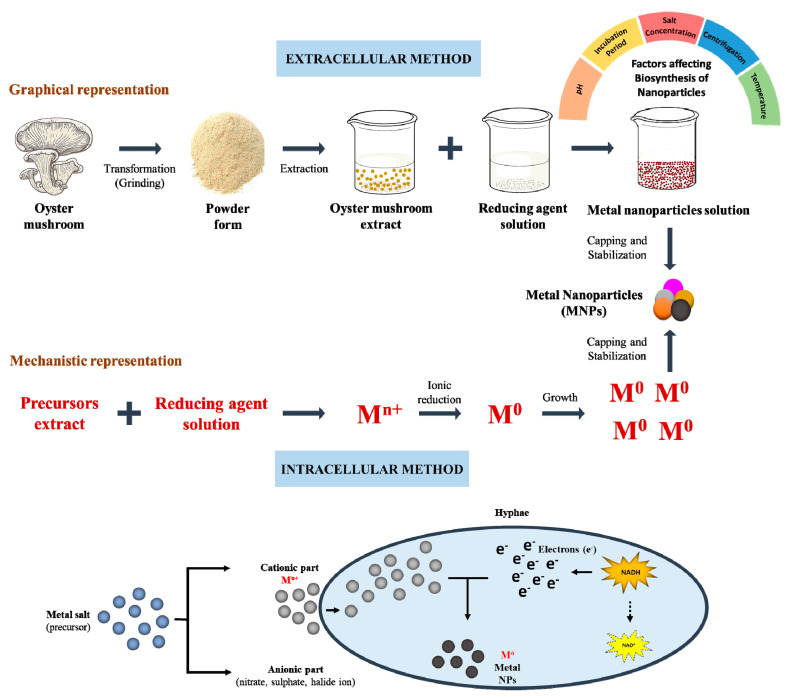
Graphical representation of green-synthesis of nanoparticles from *Pleurotus* (Oyster) mushroom.

During synthesis, fungi extracts serve the function of capping and reducing agents. At the same time, the fungal mycelium exposed to the metal precursor induces fungus to liberate metabolites and enzymes for its survival [11]. Both the fruiting bodies and mycelium of the mushrooms can be utilized for the synthesis of NPs. It has been reported that the *Pleurotus* species, such as *P. ostreatus,* are capable of synthesizing NPs, both intracellularly and extracellularly, while other species, such as *P. florida*, *P. cornucopiae var. citrinopileatus*, *P. platypus*, *P. ostreatus*, *P. sajor-caju*, *P. eous*, and *P. djamor var. roseus* synthesize NPs extracellularly [32,33,34,35,36,37]. Synthesis of *Pleurotus* derived metal NPs is shown in Table 2 and Figure 1.

### 2.1. Intracellular Method

This method includes synthesis of NPs inside the fungal cells by transporting ions during the exposure of enzymes [62,63]. First, the mycelia cultures are treated with a metal precursor and then they are incubated in the dark for 24 h. For intracellular identification, mycelia are resuspended in phosphate buffer saline (PBS, pH 7.4) and homogenized with a sonicator. NPs formed by the intracellular technique have a smaller size when compared with the NPs fabricated by the extracellular method [64,65]. Nucleation of particles inside the fungus could be the cause behind the variation in sizes. This technique is slower when compared with the extracellular method for synthesizing metal NPs [64]. As the NPs synthesis starts within the cell, their downstream processing becomes complicated, increasing cost of synthesizing NPs [66,67,68]. However, this type of synthesis technique is suitable for making composite films [69].

### 2.2. Extracellular Method

The extracellular synthesis method is a facile and cost-effective approach that involves the treatment of fungal biomolecule aqueous filtrate with a metal precursor, where these metal ions are adsorbed on the surface of the cells [31,70,71,72,73]. In this technique, downstream processing is not required, which makes this approach more effective in comparison to the intracellular method. Therefore, the extracellular approach is predominantly used for NP synthesis [74]. Extracellular metabolites synthesized by fungi play a crucial function in their survival when exposed to various environmental stresses, such as temperature variations, toxic materials (e.g., metallic ions), and predators [75]. Moreover, this synthesis method shows the capability of immobilization of metallic ions in a suitable carrier [69].

The accepted mechanism for the metallic NP synthesis is the enzymatic reduction via enzyme reductase, within the fungal cell or on the cell membrane [76]. This probable mechanism proposes fungus-mediated NP synthesis, i.e., the action of electron shuttle quinones, nitrate reductase, or by both. It is observed that, in bacteria and fungi, mainly two forms of enzymes: (1) nitrate reductase, and (2) α-NADPH-dependent reductases, are responsible for the metal and metal oxide NP synthesis [69]. Extracellularly synthesized NPs were stabilized by the enzymes and proteins formed by the fungi. Moreover, it has been observed that high molecular weight protein is associated with the synthesis of NPs, such as NADH-dependent reductase [69]. Furthermore, the phytochemicals found in plants play a vital role in the bioreduction of NPs [76]. In the mushroom extract of *Pleurotus* spp., phytochemicals, including alkaloids, saponins, anthraquinones, flavonoids, tannins, and steroids are present [14].

## 3. Different Types of Nanoparticles Derived from Oyster Mushroom

### 3.1. Silver Nanoparticles (AgNPs)

AgNPs play a significant character in the areas of biological and medical sciences. These NPs could be synthesized by various methods, such as physical, chemical, ionizing radiation methods, etc. [70]. However, all of these methods possess potential drawbacks; particularly, the chemicals utilized in AgNP synthesis through wet chemistry routes are less eco-friendly, expensive, and have high toxicity [46,77,78]. However, fabrication of AgNPs by green synthesis methods can be a better alternative as it is cost effective, non-toxic, and ecologically safe than the other synthesis methods [79]. Various studies on the biosynthesis of AgNPs using powdered basidiocarps and mycelia of different oyster mushroom species, such as *P. ostreatus, P. sajor-caju, P. florida, P. cornucopiae var. citrinopileatus, P. giganteus, P. platypus,* and *P. eous* have been reported. These basidiocarps and mycelia were soaked in distilled water, boiled, and then filtered [23,24,25,26,27,28,29,30,31,32,33]. The filtrate was freeze-dried to prepare aqueous extract. Various concentrations of this aqueous extract were incubated with AgNO_3_ solution to synthesize AgNPs by the reduction of Ag^+^ ions to Ag° (metal). Unboiled mycelia extract of *Pleurotus* has also been used to synthesize AgNPs [38]. In their report, they crushed the fruiting bodies and mixed them with deionized water. The content was filtered with filter paper, and then the filtrate was used to synthesize AgNPs, with a size of 6–10 nm, with a spherical shape. Moreover, the synthesized AgNPs were further assessed for antibacterial potential against *Escherichia coli* and *Staphylococcus aureus*.

Synthesis of AgNPs was carried using *P. tuber-regium* mushroom extract and 1 mM AgNO_3_ solution. The mixture of solutions was stirred at 90 °C for 2 h. Cubical and spherical shaped AgNPs, with an average size of 50 nm, were obtained as a black powder [61]. Debnath et al. synthesized spherical shaped AgNPs with the help of aqueous extract of mushroom (5 mL) and mixed with 95 mL silver nitrate (1 mM, AgNO_3_) solution to reduce Ag^+^ to Ag^o^. This solution was kept in an incubator for 3 days at 37 °C, resulting in color change from light yellow to yellowish-brown. The obtained AgNPs were crystalline with a size ranging from 5 to 25 nm. Authors evaluated the antibacterial activity of AgNPs against *E. coli*, *B. subtilis*, *P. aeruginosa*, and *S. aureus* [51]. Similarly, the synthesis of predominantly spherical shaped AgNPs with a size ranging from 2 to 100 nm was carried by various researchers using mushroom extract and AgNO_3_ solution [34,37,52,57,80].

### 3.2. Gold Nanoparticles (AuNPs)

AuNPs synthesis was performed by using edible *P. florida* mushroom by the photo-irradiation method, and evaluated for anticancer potential against A-549, HeLa, K-562, and MDA-MB cell lines. Initially, 5 g of fresh biomass of *P. florida* mushroom was washed with deionized water and then cut to small pieces. Later, the chopped pieces were added in 500 mL of double-deionized water, under stirring, for half an hour. These contents were then incubated overnight. That content was then filtered via filter paper. Later, the filtrate of mushroom was used to reduce Au^+^ into Au° in the presence of bright sunlight to form spherical to triangular-shaped AuNPs in the range of 10–50 nm [48].

### 3.3. Zinc Sulfide Nanoparticles (ZnS) and Zinc Oxide Nanoparticles (ZnO)

ZnS NPs were fabricated using *P. ostreatus* extract, ZnCl_2_, and Na_2_S solution as the precursor material [55]. Firstly, small pieces of mushrooms were boiled and filtered. Then, different concentrations of the resultant filtrate were mixed with aqueous solutions of ZnCl_2_ and Na_2_S solution, and resulting solutions were dried at 120 °C for 2 h. Here, the resultant filtrate was used as a stabilizing (as well as a capping) agent for the fabrication of spherical shaped ZnS NPs. Obtained ZnS NPs was highly crystalline with sizes varying from 2.30 nm to 4.04 nm. The author observed that the diameter of those spherical ZnS NPs was decreasing with the increase in extract amount [55]. ZnONPs were synthesized by using *P. djamor* extract, 20 mL of mushroom extract added into 80 mL of Zn (NO_3_)_2_. The 5H_2_O (5 mM) solution was continuously mixed for 24 h at room temperature until the color transformed into light pink, which confirmed the synthesis of ZnONPs [43].

### 3.4. Cadmium Sulfide Nanoparticles Quantum Dots (CdS QDs)

In contrast to traditional fluorescent organic dyes and green fluorescent proteins, CdS QDs seem to be superior as they overcame the limitations associated with different factors, such as spectral overlapping, weak signal intensity, and photobleaching [81]. The multiple characteristics of QDs are high photostability, symmetric, slow decay rates, fine emission spectra, wide absorption cross-sections, and broad absorption spectra. The emission color of QDs depends upon their size and surface chemistry; chemical composition used can be altered from the UV to visible or near NIR wavelengths. The increasing interest in the use of CdS QDs is because they act as luminescent probes and labels for biological imaging, disease diagnosis, and molecular histopathology. The studies revealed that the QDs derived from plants did not aggregate [82]. Borovaya et al. synthesized CdS NPs with the help of aqueous extract of roots of *Linaria maroccana*, CdSO_4_, and Na_2_S. First, the mixture solution was incubated for 4 days at 28 °C resulting in the formation of the clear homogeneous solution with a bright yellow color. This indicates the formation of CdS NPs, which are water-soluble and spherical, with sizes of 5–7 nm [82]. In 2015, again biosynthesis of luminescent CdS NPs using mycelium of *P. ostreatus*, CdSO_4_ and Na_2_S. In brief, CdSO_4_ solution was mixed with mycelium followed by the incubation for 10 days at 26 °C, followed by the addition of Na_2_S solution. Obtained NPs were spherically shaped, having the size in the range of 4 to 7 nm. In particular, cadmium sulfide QDs are highly useful in investigating the biomolecules interaction and cellular signaling pathway with the help of fluorescent microscopy [81].

### 3.5. Titanium Dioxide Nanoparticles (TiO_2_)

TiO_2_ NPs were synthesized by using edible *P. djamor* mushroom and evaluated for anticancer potential against A-549 (human lung carcinoma) cell lines, as well as for larvicidal and bactericidal activity. Initially, 10 g of fresh biomass of *P. djamor* mushroom was washed with deionized water for 10 min and then cut to small pieces. Later, the chopped pieces were added in 100 mL of double- deionized water, boiled at 60 °C for 15 min, and then filtered. Then, 20 mL of filtrate was added to 80 mL of TiCl_4_ (5 mM) solution, stirred for 2 h, and kept to room temperature for 20 min until the color changed to brown. The intensity of the color of the extract was determined at the wavelength of 345 nm. The synthesized TiO_2_ NPs formed, spherical in shape, with sizes of 31 nm [44].

### 3.6. Synthesis of Other Nanoparticles

#### 3.6.1. Iron Nanoparticles (FeNPs)

FeNPs were intracellularly synthesized by using hypha of *Pleurotus* sp. The reduction process is involved in uptake of FeNPs via the fungal cell membrane, in which reduction of ferric ion (Fe^+3^) to ferrous ion (Fe^+2^) takes place. The reduction process is involved during the iron uptake by fungi [83].

#### 3.6.2. Selenium Nanoparticle (SeNP)

SeNPs were synthesized via mushroom polysaccharide-protein complexes (PSPs) isolated from *P. tuber-regium* sclerotia. These NPs have anticancer activity, excellent bioavailability, and low toxicity. SeNPs have been recorded for inhibiting the proliferation of human breast carcinoma MCF-7 cells by apoptosis; results obtained from the study revealed that cytotoxicity was cancer-specific [84]. PSP–SeNPs have the efficiency to enhance the reactive oxygen species (ROS) generation, and inhibit dose dependently the growth of MCF-7 human breast carcinoma cells, through induction of apoptosis, with the involvement of Poly (ADP-ribose) polymerase (PARP) cleavage and caspase activation. The size of PSP–SeNPs with an average diameter < 50 nm spherical in shape [85]. The synthesis of SeNPs from *P. ostreatus* extract have been reported for in vitro anticancer activity [86].

#### 3.6.3. Copper Nanoparticles (CuNPs)

Monodispersed copper nanoparticles (CuNPs) were synthesized from aqueous fermented fenugreek powder (FFP), polysaccharides, such as chitosan, sodium alginate, citrus, and pectin, with the help of fungal strains of *P. ostreatus*, under the exposure of gamma radiation. The CuNPs synthesized have size ranges from 25.0 to 36.0 nm. Because of the stability and the minute sizes, these synthesized CuNPs show antioxidant and antimicrobial activity, and were found beneficial in cosmetics, medical, pharmaceutical, and industrial applications [87].

## 4. Applications of Pleurotus Derived Nanoparticles

**Figure 2:**
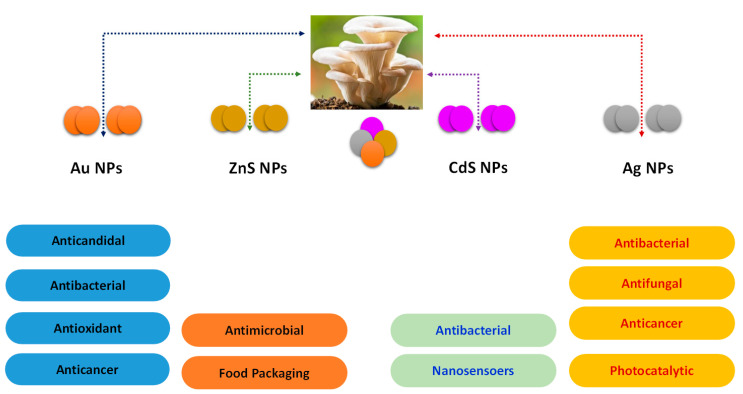
Schematic diagram showing the applications of oyster mushroom derived nanoparticles.

Different types of metal NPs synthesized from oyster mushrooms are discussed above. Recently, they have been considered as valuable in various fields of medicine and industries. Schematic representation of different applications of metal NPs derived from *Pleurotus* is summarized in Figure 2.

### 4.1. Antimicrobial Activity

In the past few decades, the microbial infection has become a major health issue across the world, due to its continuously evolving nature and ability to develop resistance against the existing regime [64]. Therefore, search for a strong alternative candidate that can kill or inhibit multidrug resistance microbes is needed [88]. Metal nanoparticles (MNPs) are known to possess potent antimicrobial activity against a wide variety of microbes, including bacteria (Gram-negative and Gram-positive) and fungi, via their photodynamic effects and strong oxidative stress [89]. Furthermore, the direct physical contact of MNPs to bacterial membrane results in the release of intercellular material, loss of cell membrane integrity, and cell death [88]. In literature, several reports are offered on MNPs’ (AgNPs, AuNPs, ZnSNPs) exhibiting bactericidal activity. However, only a few reports focus on the support of their antifungal and antibacterial activity with appropriate mechanisms [88,89,90,91]. Moreover, many reports available in literature that favor the mechanism involved in bacterial and fungi cells are almost similar.

In the year 2009, Nithya and Ragunathan synthesized AgNPs (5–50 nm, spherical) derived from *Pleurotus sajor-caju* fungi as the starting material and evaluated their bactericidal activity against the both Gram-negative (*Pseudomonas aeruginosa*, *E. coli*) and Gram-positive (*S. aureus*) bacteria. Authors claimed that the inhibitory action of AgNPs was attributed to the generation of Ag^+^ ions by NPs that resulted in DNA damage, protein denaturation, enzymes inhibitions [32]. Two years later, in 2011, Bhat et al. also fabricated AgNPs (20 ± 5 nm, spherical) using *P. florida* mushroom, and studied their antibacterial activity against *S. aureus, Salmonella typhi, Providencia alcalifaciens,* and *Proteus mirabilis*. The prepared NPs show higher activity against the Gram-positive microbes than Gram-negative, especially in the case of *P. mirabilis* [33]. A similar kind of study was presented by Shivashankar et al. (2013) using AgNPs derived from *P. pulmonarius*, *P. djamor*, and *Hypsizygus (pleurotus) ulmarius* as the precursors [40,41].

Yehia and Sheikh (2014) synthesized AgNPs (4–15 nm, spherical) derived from *P. ostreatus* extracted via the green synthesis route and evaluated their antifungal activity toward the various *Candida* species, i.e., *C. tropicalis*, *C. albicans*, *C. parapsilosis*, *C. krusei*, and *C. glabrata*. The (minimum inhibitory concentration) MIC (IC_80_) results demonstrated that AgNPs showed higher toxicity against all of the *candida* species (5–28 μg/mL) than the amphotericin B (5–8 μg/mL) and fluconazole (13–33 μg/mL) [36]. The tiny size and capping ability of the bioactive white NPs derived from *P. tuber-regium* extract and silver nitrate showed higher therapeutic efficacy against the various diseases and disorders [61]. Devi and Joshi (2015) synthesized AgNPs derived from three different endophytic fungi, i.e., *Aspergillus niger*, *Aspergillus tamarii*, and *Penicillium ochrochloron* isolated from the ethnomedicinal plant *Potentilla fulgens* leaves via the green synthesis method. The electron microscopy results revealed that all of the AgNPs derived from different fungi were spherically shaped. However, NPs synthesized from *A. tamarii* showed the smallest size (~3.5 nm) than *A. niger* (~8.7 nm) and *P. ochrochloron* (~7.7 nm), respectively [74]. In the year 2018, Bawadekji et al. fabricated Au NPs (~22.9 nm, spherical) from *P. ostreatus* extract and evaluated their antimicrobial activity toward the bacteria *Enterococcus faecalis*, *E. coli*, *Klebsiella pneumonia*, *S. aureus*, *P. aeruginosa*, and *C. albicans*. The results demonstrated that synthesized NPs showed significant toxicity against *C. albicans*, *P. aeruginosa,* and *S. aureus*. In contrast, no toxicity was observed in the case of *E. faecalis*, *E. coli*, and *K. pneumonia* [54].

**Figure 3:**
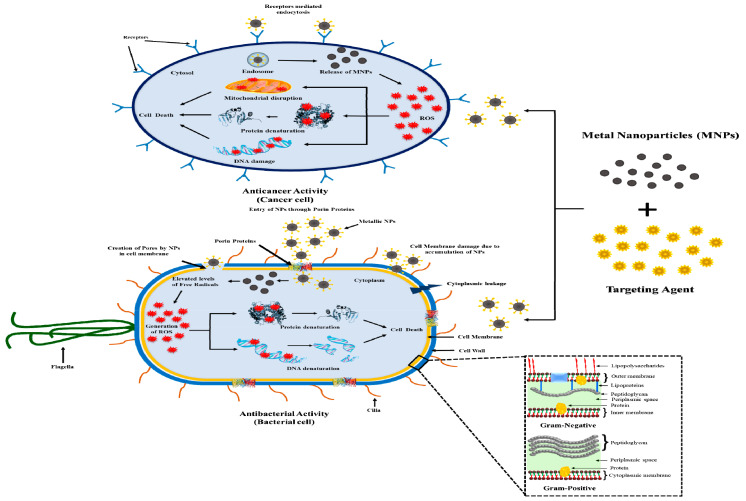
Graphical representation of mechanism showing anticancer and antimicrobial activity.

Acay and Baran (2019) reported the green synthesis of AgNPs derived from *Pleurotus eryngii* (PE) extract and their antimicrobial activity against the various human pathogen microorganisms, such as *E. coli*, *S. aureus*, *Streptococcus pyogenes*, *P. aeruginosa*, and *C. albicans*. The authors used drug vancomycin, colistin, and fluconazole as the control over the gram-positive, gram-negative, and fungus microorganisms. The observed MIC values for *S. aureus*, *E. coli*, *S. pyogenes*, *C. albicans*, and *P. aeruginosa* were 0.035, 0.07, 0.018, 0.07, and 0.035 mg/L, respectively. Authors claimed that AgNPs derived from (PE) *Pleurotus eryngii* extract could be used as a better alternative, as an antibiotic, compared to the other silver nitrates and antibiotics [45]. After that, Debnath et al. (2019) also fabricated AgNPs from *Pleurotus giganteus* and analyzed their antibacterial activity [51]. The TiO_2_ NPs mediated from extract of *P. djamor* exhibited significant bactericidal activity against human pathogenic bacteria with maximum zone of inhibition *P. fluorescens* (33 ± 0.2 mm), *Corynebacterium diphtheria* (32 ± 0.1 mm), *S. aureus* (32 ± 0.4 mm), and showed higher levels of the inhibitory effect [44]. The *P. djamor* ZnONPs showed a maximum zone of inhibition against *C. diphtheriae* (28.6 ± 0.3 mm), *P. fluorescens* (27 ± 0.5 mm), and *S. aureus* (26.6 ± 1.5 mm) [43]. The general mechanism of microbial cell death is summarized, as below and in Figure 3.

#### 4.1.1. Antimicrobial Mechanisms

##### Physical Destruction

In this case, positively charged metal NPs can easily bind to the negatively charged components (i.e., porins, peptidoglycans, and proteins) of the cell membrane via electrostatic interaction, leading to damage of bacterial/fungi membrane, intercellular leakage and, finally, cell inhibition [92].

##### Oxidative Stress

Another primary mechanism for the antimicrobial activity is based upon the occurrence of oxidative stress, either in the presence of light or under dark conditions. In the microbial cell, the metal NPs can generate ROS, such as •OH and •O2, leading to protein denaturation, DNA damage, enzyme activation, ribosome disassemble and, finally, cell death [92,93]. Furthermore, metal NPs can also act as photoabsorber material upon excitation of light (most often NIR), resulting in cell death. The photothermal effect comes in origin when the emitted electrons from a higher energy state returns to a low energy state, and release their energy in the form of heat and vibrational energy [94].

### 4.2. Antioxidant Activity

**Figure 4:**
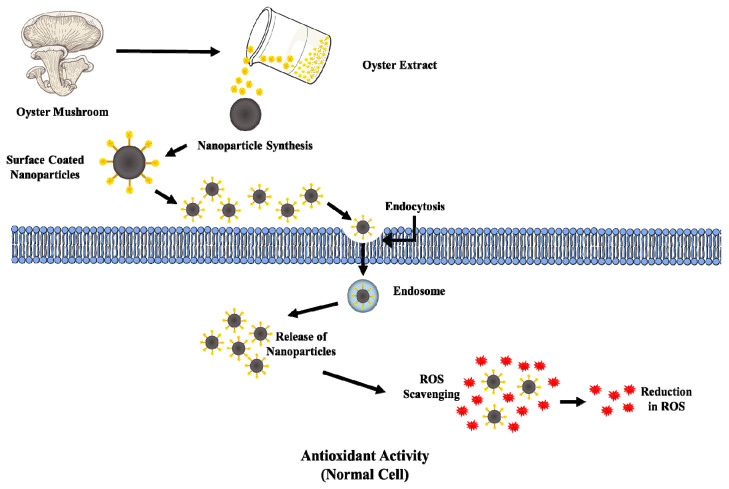
Schematic diagram of the antioxidant mechanism.

In the human body, excessive reactive free radicals are formed from various sources, such as low diet, mental stress, smoking, and other ailments [95]. Metal NPs exhibited profound antioxidant activities in both intracellular and extracellular environments, as summarized in Figure 4.

In the year 2012, Adebayo et al. synthesized metabolite derived from *P. pulmonarius* extract and evaluated their radical scavenging ability via α, α-diphenyl-β-picrylhydrazyl (DPPH) radical scavenging assay and the β-carotene-linoleate model method. The metabolite derived from *P. pulmonarius* extract showed dose-dependent radical scavenging activity. It was found that the existence of glutathione, ascorbic acid, cysteine, tocopherol, polyhydroxy compounds, and aromatic amines in metabolite reduces and decolorizes the violet color of DPPH via hydrogen transferability. Authors claimed that, at a concentration of 2 mg/mL, metabolite showed butylated hydroxyanisole (BHA) (75%), LAU 09 (80%), and α-tocopherol (90%) of inhibition, which attributes to of the presence of phenolic compounds in the extract [14]. A few years later, in 2017, Madhanraj et al. synthesized gold (Au) and silver (Ag) nanoparticles derived from edible mushroom (basidiomycetes) and studied their antioxidant activity via various radical scavenging assays. Both the prepared NPs (Au & Ag) showed significant antioxidant activity in a cell-free system [39]. Acay and Baran (2020), synthesized *P. eryngii* AgNPs and evaluated their radical scavenging ability via DPPH, chelation of ferrous ions reducing power, and the β-carotene-linoleate model method, and found that, at a concentration of 10 mg/mL, antioxidant activities were 85%, 82%, and 77%, respectively [96]. Zinc plays a role in protecting cells from oxidative stress and acts as an antioxidant. The ZnONPs derived with the help of *P. djamor* possess strong antioxidant properties (DPPH 59%, H_2_O_2_ 59.65%, and ABTS 59.30%), with IC_50_ values of 428.35 µg/mL, 417.22 lg/mL, and 500 lg/mL), respectively [43].

Two possible primary mechanisms for the antioxidant activity are; (i) hydrogen atom transfer, and (ii) single electron transfer [92]. Excessive free radicals could be neutralized or terminated via donating a hydrogen atom that includes total oxyradical scavenging capacity assay, inhibition of induced low-density lipoprotein oxidation, oxygen radical absorbance capability, and radical-trapping antioxidant parameters [78]. On the other hand, the single-electron transfer involves the reduction of compounds, such as radicals, metals and carbonyls by transferring one electron, including change in the color when the compound is reduced, such as Ferric Reducing Antioxidant Potential (FRAP), 2,2-diphenyl-1-picrylhydrazyl radical (DPPH) [92]. However, in the intracellular level, metal NPs enter inside the cells via endocytosis and decrease the ROS levels generated by any probe; for example, 2, 7′-dichlorodihydrofluorescein diacetate (DCFDA) [92].

### 4.3. Anticancer Activity

In addition to antibacterial and antioxidant activity, metal NPs derived from fungi and other sources have been known to possess outstanding anticancer activity because of their profound ROS generation ability under the dark and light exposure [92,97,98,99]. Sankar et al. (2013) studied the anticancer activity of AgNPs (~136 nm) derived from *Origanum vulgare* extract against the human lung epithelial cells (A549 cells). AgNPs exhibited dose-dependent toxicity against the A549 cells by 85% inhibition at the dose of 500 μg/mL [100]. Bhat et al. (2013) fabricated Au NPs (12–15 nm, spherical) derived from *P. florida* mushroom extract via the photo-irradiated method and evaluated their anticancer activity against the A-549, MDA-MB, HeLa, and K-562 cell lines. The prepared AuNPs showed concentration-dependent activity against all cell lines in between 10 and 30 μg/mL [48].

Gliga et al. (2014) attempted to understand the coating and size-dependent toxicity of the AgNPs toward the human lung cells (BEAS-2B cells) with an appropriate mechanism. The results confirmed that prepared NPs with size <10 nm showed the highest toxicity against the BEAS-2B cells, which attributes for its aggregation in cell medium, intracellular localization, cellular uptake, and formation of Ag ions intracellularly. However, it is confirmed that all of the AgNPs showed toxicity against BEAS-2B cells via an increase in overall DNA damage within 24 h [101]. In the same year, Yehia and Sheikh (2014) used *P. ostreatus* derived AgNPs (4–15 nm, spherical) as an anticancer agent against the MCF-7 cells. The prepared AgNPs showed dose-dependent cell inhibition ranging from 5% to 78% at concentration 10 to 640 μg/mL [36].

Similarly, in the year 2015, Ismail et al., fabricated AgNPs derived from *P. ostreatus* extract and studied their anticancer effect against the HepG2 and MCF-7 adenocarcinoma cancer cell lines. The authors claimed that NPs induced cytotoxicity toward cancer cells attributes for the formation of ROS species, apoptosis, necrosis, and cell death. ROS are the highly reactive species that result in oxidative damage of proteins, DNA, and induce mitochondrial dysfunction, as summarized in Figure 3 [102]. Similarly, Raman et al. (2015) used *P. djamor* var. roseus derived AgNPs as anticancer agent toward the human prostrate carcinoma PC3 cells [42]. In the year 2013 and 2014, Priyaragini and Kim et al. demonstrated that metal NPs are harmless at a lower concentration and may be lethal at a higher dose toward normal healthy cells. Many reports revealed the biosynthetic routes to synthesize AgNPs as an anticancer agent against various cell lines. However, AgNPs synthesized using green methods also showed a sort of cytotoxicity against tumor cells [103,104]. Even the extensive use of artificial AgNPs has been already reported, but still, there are limited studies to regulate the cytotoxic effects of AgNPs [36]. Studies on *P. eryngii* (PE) AgNPs showed cytotoxic activity of HeLa with maximum inhibitory effect 73.46% at 60 μg/mL concentration, PC-3 99.02% at 10 μg/mL and MCF-7 cells 93.89% at 20 μg/mL concentration with IC_50_ values of 46.594, 2.185, and 6.169 μg/mL, respectively, during a 24-h incubation period [90]. Chaturvedi et al. (2020) studied cytotoxic activity and revealed that the AgNPs and AuNPs mediated from *P. sajor-caju* extract (PS) showed effective results against HCT-116 cancer cell line. HCT-116 cancer cells viability showed inhibition by *P. sajor-caju* extract, Au NPs as well as Ag NPs showing IC_50_ value of 60, 80, and 50 µg/mL respectively. The study revealed that the green synthesized AgNPs showed high antiproliferative activity in contrast to other PS extract and Au NPs, and the reason behind the mechanism was due to the generation of more ROS, leading to oxidative stress, resulting in undeviated damage of protein functionality and integrity [60]. The anticancer activity of TiO_2_ NPs showed potential toxic effect against human lung cancer (A549) cell lines with maximum inhibited growth of 64% at concentration of 100 µg/mL, after 24 h of exposure [44]. The anticancer activity evaluated from *P. djamor* ZnONPs showed potent inhibitory on A549 cancerous cells with (LC50(Lethal concentration required to kill 50% of population) value as 42.26 µg/mL) in a dose-dependent manner [43].

### 4.4. Histopathological Study and Larvicidal Activity

The histopathological profile of TiO_2_NPs mediated from a *P. djamor* extract treated mosquito (*Aedes aegypti and Culex quinquefasciatus*) resulted in the complete collapse of caeca, digestive tract, and desertion of the cuticle and epithelial layer, with harsh damage to the mid-and hind-gut, muscles, as well as nerve ganglia of the brush border. The treating of TiO_2_ NPs on IVth instar larvae of *Ae. aegypti* and *Cx. quinquefasciatus* resulted in larvicidal activity with LC50 (5.88 and 4.84 µg/L) and LC90 (22.80 and 19.33 µg/L) [44]. The *Ae. aegypti* larvae treated with ZnONPs showed morphological alteration in the digestive tract, wrecked membrane, midgut, and severe damaging of the brush border, cortex with hyperplasia of gut epithelial cells, and variations in the cytoplasmic masses. The larvae of *Cx. quinquefasciatus* showed the complete putrefaction of abdominal parts, specifically in the caeca, mid-gut, and epithelial layer [43].

### 4.5. Antidiabetic Activity

The antidiabetic activity was investigated in vitro through the inhibition of α- amylase, an enzyme that digests starch. AgNPs synthesized from *P. giganteus* possess good α-amylase inhibition activity, which helps in making diabetic drugs; inhibition percentage can be increased with increasing concentration of biosynthesized AgNPs [51].

### 4.6. Removal of Dyes

El-Batal et al. (2014) extracted fungal laccase derived from *P. ostreatus* via solid fermentation. The authors demonstrated that this enzyme could be used to decolorize/degrade numerous dyes, i.e., methyl orange, trypan blue, ramazol brilliant red, and ramazol brilliant yellow with more than fifty percent decolorization in their color within 3 h, confirming the laccase degrading ability. The highest reduction was observed for the methyl orange and trypan blue. Furthermore, laccase enzyme was used to synthesize gold NPs, proving that laccase obtained from *P. ostreatus* had strong potential in many significant industrial applications, for example, in biological pretreatment processes [105,106].

### 4.7. Catalytic Activity

The recent use of the 4-nitrophenol and derivatives in the manufacturing of insecticides, herbicides, and dyestuffs cause harm to the environment as common wastewater pollutants. Because of their high toxicity, it is challenging to eliminate these pollutants, which is a primary environmental concern. In the year 2007, Panigrahi et al. prepared citrate-capped negatively charged Au NPs (8−55 nm, spherical) for the catalytic degradation of aromatic nitro compounds. The authors claimed that the rate of the reaction rose with the rise in the loading of the catalyst, and decreased in particles size, clearly reflecting the catalytic behavior of gold nanoparticles against aromatic compounds, resulting in amino-compounds [107]. Similarly, Lim et al. (2016) prepared gold nanoparticles (AC-Au NPs, 16.88 ± 5.47~29.93 ± 9.80 nm, spherical) from *Agrostis capillaris* extract, and studied their catalytic efficacy in the presence of NaBH_4_ against the 4-nitrophenol. They demonstrated that particle size falls with the rise in extract concentration during the synthesis process. It was observed that the catalytic degradation of 4-nitrophenol rises as the particles size decreases [108].

In the same year, 2016, Rostami-Vartooni et al. developed AgNPs (8–35 nm, spherical) loaded on perlite (sheet-like) using *Hamamelis virginiana* leaf extract and evaluated their catalytic activity against the 4-nitrophenol and Congo red (CR) dye. The authors demonstrated that, with the rise in the concentration of NaBH_4_ and AgNPs/perlite, the degradation time of 4-nitrophenol decreases, respectively. The AgNPs supported on the surface of perlite facilitate the electron relay from BH_4_- to 4-nitrophenol as well as CR dye. Furthermore, they claimed that AgNPs/perlite showed high stability and could be used up to 4 times with significant degradation efficacy [109]. Later, Gopalakrishnan et al. (2017) reported the catalytic degradation of 4-nitrophenol to 4-aminophenol via NaBH_4_ in the presence of PdNPs (<20 nm, spherical) derived from seed extract of *Silybum marianum*. The total reduction action was attained within 27 min and is attributed to the relay of electrons from BH4- to 4-nitrophenol, resulting in 4-aminophenol. However, the authors claimed that no reduction was detected in the case of bare NaBH_4_ [110]. A similar kind of 4-nitrophenol reduction was also performed by Sen et al. (2013) using *P. florida* derived AuNPs and NaBH_4_ [49].

### 4.8. Food Packaging and Preservation

Biocompatible fabricated zinc NPs might be efficiently applied in the biomedical and food packaging fields. The potential antimicrobial action of mushroom against many foodborne bacteria, such as *Escherichia coli*, *Streptococcus faecalis*, *Bacillus subtilis*, *Micrococcus luteus*, and *Listeria innocua* could be considered as a boon for the food industry since, by using metal NPs, the contamination of foodstuffs can be avoided, besides for long-time preservation [55,111].

## 5. Conclusions

Nanomaterials derived from oyster mushrooms have been found to possess great potential over a wide range of applications, especially in the biomedical field. In the present review, we discussed the progress of research, to date, on metal nanoparticles and other nanomaterials derived from oyster mushrooms, regarding their synthesis and applications, particularly in the areas of antimicrobial, larvicidal, antioxidant, anticancer, and catalysis. Generally, AgNPs derived from *Pleurotus* spp. have a higher synthesis and biomedical applications among mushrooms. The importance of derived nanoparticles is due to their unique characteristics, such as cost effective, crystalline nature, nanosize, and non-hazardous nature. Mainly there are two well-known methods of synthesis, i.e., intracellular and extracellular. It is noticed that nanomaterials derived from oyster mushrooms showed profound applications in the areas of biomedicine and catalysis, but some areas of research are needed to be addressed, which are as follows:

- To date, oyster mushroom derived NPs are not directly applied to the live samples. Hence, the progress can be made in this direction.
- Available literature provides evidence that considerable work has been carried out for ascertaining the efficacy of oyster mushroom derived NPs under in vitro conditions against the various cancer cell lines. As less information is available regarding in vivo studies, there is a need for further exploration.
- More studies are needed to define oyster mushrooms that can be genetically engineered to produce more enzymes primarily involved in NP synthesis, and to expand the knowledge and functions of nanomaterial, so that significant achievements could be attained in the fields of medicine, electronics, cosmetics, agriculture, the environment, and many more.

## Figures and Tables

**Table 1:** Geographical Distribution of *Pleurotus* spp.

Species	Geographical Distribution	Optimum Growth Temperature °C	Reference
*Pleurotus citrinopileatus*	Russia, China, Japan	21–29	[8,9]
*P. cornucopiae*	Europe, USA, and Mexico	>25	[8,9]
*P. djamor*	Tropical region, Indonesia, Malaysia, Japan, Mexico	21–35	[8,9]
*P. eous*	Sub-tropical part of the world	23–28	[8,9]
*P. eryngii*	Europe, Asia, Africa	20–25	[8,9]
*P. flabellatus*	India, Mauritius	25–28	[8,9]
*P. florida*	Hungary, Kenya	20–28	[8,9]
*P. giganteus*	Thailand, Sri Lanka	15–35	[8,10]
*P. ostreatus*	Widespread around the world	18–22	[8,9]
*P. sajor-caju*	Kenya, India, Philippines, Australia, Mauritius	20–28	[8,9]
*P. tuber-regium*	Africa, Australia, Asia	25–30	[8,9]
*P. platypus*	India	15–25	[8,9]
*P. pulmonarius*	Warm tropical area	20–28	[8,9]

**Table 2:** Varieties of *Pleurotus* spp. and their nanoparticles.

Species	Types of Nanoparticles Synthesize and Their Size (nm)	Chemical Used	Reaction Time (hour)	Reducing Agent	Stabilizing Agent	Specific Temperature (°C)	Morphology	References	
*Pleurotus citrinopileatus*	Ag, 6–10	AgNO_3_	24	mushroom extract, Nitrate	mushroom extract	60	spherical	[38]	
*P. cornucopiae (citrinopileatus)*	Ag, 20–30	AgNO_3_	24	aqueous extract	aqueous extract	25	spherical	[37]	
*P. cystidiosus*	Ag, 2–100	AgNO_3_	24	aqueous extract	aqueous extract	25	ND	[39]	
*P. cystidiosus*	Au, ND	HAuCl_4_	24	aqueous extract	aqueous extract	29	ND	[39]	
*P. djamor*	Ag, 5–50	AgNO_3_	48, 24	aqueous extract	aqueous extract	RT	spherical	[40,41,42]	
*P. djamor*	ZnO, 70–80	Zn(NO3)2.5H2O	24	ND	ND	RT	spherical	[43]	
*P. djamor*	TiO2	TiCl4	20 min	aqueous extract	aqueous extract	RT	spherical	[44]	
*P. eryngii*	Ag, 18.45	AgNO_3_	5 days	aqueous extract	aqueous extract	RT	spherical	[45]	
*P. flabellatus*	Ag, 2–100	AgNO_3_	24	aqueous extract	aqueous extract	25	ND	[39,40,41]	
*P. flabellatus*	Au, ND	HAuCl_4_	24	aqueous extract	aqueous extract	29	ND	[39]	
*P. florida*	Ag, 20	AgNO_3_	Overnight; 72	aqueous extract	aqueous extract	RT	spherical	[33,34,40,41,46,47,48]	
*P. florida*	Au, 2–14	HAuCl_4_	1.5	aqueous extract, glucan	glucan	70	spherical	[49]	
*P. florida*	Au, 20	HAuCl_4_	24	aqueous extract	aqueous s extract	RT	spherical	[50]	
*P. giganteus*	Ag, 5–25	AgNO_3_	3 days	aqueous extract	aqueous extract	37	spherical	[51]	
*P. ostreatus*	Ag, 4,28,50	AgNO_3_	24; 72; 1	aqueous extract; mushroom broth	aqueous extract	28; 75	spherical;	[36,39,52,53]	
*P. ostreatus*	Au, 22.9	HAuCl_4_	24 h	aqueous extract	aqueous extract	29	spherical	[39,54]	
*P. ostreatus*	ZnS, 2–5	ZnCl_2_	Over night	mushroom	mushroom extract	70	spherical with crystalline	[55]	
*P. ostreatus*	Zn, 15	ZnS-N_3_	1	aqueous extract	aqueous extract, sodium azide	4	uniform	[56]	
*P. platypus*	Ag, 0.56µm	AgNO_3_	72	aqueous extract	aqueous extract	37	spherical	[34]	
*P. pulmonarius*	Ag, 2–100	AgNO_3_	24	aqueous extract	aqueous extract	25	ND	[40,41]	
*P. pulmonarius*	Au, ND	HAuCl_4_	24	aqueous extract	aqueous extract	29	ND	[39]	
*P. sajor-caju*	Ag, 5–50	AgNO_3_	48	aqueous extract	aqueous extract	25	spherical	[32,57,58,59,60]	
*P. sajor-caju*	Au, 16–18	HAuCl_4_·3H_2_O	Over night	aqueous extract	aqueous extract	RT	spherical	[60]	
*P. tuber-regium*	Ag, 50	AgNO_3_	2	aqueous extract	aqueous extract	80	spherical and cubical	[61]	

Note: RT—room temperature; ND—not detected.
